# Study on the relationship between buyer market power, spatial spillover effect, and profit in China's pharmaceutical industry

**DOI:** 10.3389/fpubh.2025.1683074

**Published:** 2025-11-14

**Authors:** Yu Guanyi, Wu Lei, Chen Heng, Ding Zhengliang

**Affiliations:** 1 School of Economics and Management, Harbin Engineering University, Harbin, China; 2 Decision-Making Consultation Department, Party School of Heilongjiang Provincial Committee of Communist Party of China (Heilongjiang Academy of Governance), Harbin, China

**Keywords:** buyer market power, seller countervailing power, profit, spatial Durbin model, spatial spillover effect

## Abstract

Previous studies have extensively explored industrial innovation, but the impact of downstream buyer market power on upstream industries remains underexamined. Using a spatial Durbin model (SDM) and data from China's medical and pharmaceutical sectors (2001–2021), we analyze how buyer market power affects pharmaceutical industry profitability. Key findings include: (1) local buyer market power reduces local pharmaceutical profitability and may also negatively affect other regions through spatial spillover, though this spillover effect is weak. (2) Stronger regional economic ties amplify the impact of local medical industry power on pharmaceutical profitability. (3) Supplier countervailing power can mitigate the negative effects of buyer power on pharmaceutical profits. (4) Buyer market power significantly harms pharmaceutical profitability in western China and low capital-intensity sectors, but not in eastern or central regions and high capital-intensity sectors. (5) Asset specificity intensifies the negative impact of buyer power, while larger firm size helps reduce it. (6) Buyers can lower pharmaceutical profitability by reducing R&D investment. This study contributes to industrial organization theory by revealing how downstream buyer power affects upstream profitability. It expands empirical methods by incorporating spatial econometrics and offers policy suggestions for improving pharmaceutical industry performance from a vertical chain perspective.

## 1 Introduction

Buyer power refers to the market power possessed by the buyer in a market transaction. Galbraith (1) expressed the buyer power for the first time as the market power owned by the buyer, being able to countervail with the manufacturer. He believed that when the seller occupies a dominant position in the industrial chain, the buyer can rely on the counterbalance power to pass on the cost savings to consumers, finally reducing the product price and improving consumer welfare. Galbraith (1) affirmed the emergence and effect of buyer counterbalance power.

In the previous studies on the buyer counterbalance power the implied background of the industrial chain is that the buyer power is relatively small and the seller power is dominant, thus it is called “buyer counterbalance power.” As the market power from of downstream customers increased gradually, the position of buyers and sellers in market transactions shifted. In this situation, countervailing power of buyers to only necessary to counter dominant supplier market power, but previous studies do not consider what the impact on social welfare (however defined) will be if buyer market power becomes dominant. Theoretically, in the case of the industrial chain dominated by downstream buyer, the effect of the buyer power may be the same as the effect of counterbalance power. However, it may not be the same, because the balance of the power between buyers and sellers shifted, and buyer power has become a new kind of power.

Relying on the realistic background of the Chinese market, we choose the pharmaceutical industry to carry out the research, a typical industry with prominent buyer power. By virtue of professional diagnosis, treatment knowledge and services, doctors extend their market power in the diagnosis and treatment market to the drug sales market. In this way, hospitals turned into very powerful negotiating buyers, they tend to possess a strong and indisputable bargaining power when facing pharmaceutical companies. Compared with other industries, the pharmaceutical industry is a typical industry where buyer power is prominent. Therefore, it can be used as the appropriate research object of buyer power effect.

The purpose of this paper is to empirically explore the influence of buyer power on the supplier's profit in the case of the new buyer-led vertical relationship when the position of the buyer and the seller changes. This paper adds to or expands on existing literature on buyer countervailing or counterbalance power in pharmaceutical markets. This study confirms that the buyer power faced by pharmaceutical enterprises (the large buyer counterbalance power developing to a certain stage) does exist and is universal. When the position of the buyer and the seller changes, in the case of the new buyer-led vertical relationship, the distortion of the vertical relationship caused by the buyer power will have a negative impact on the upstream industry. This effect is in contrast to the beneficial effect of buyer counterbalance power (1).

Based on the spatial econometric model, this paper innovates the research method of vertical relationship, which provides evidence of the interaction between upstream and downstream industries. Specifically, from the perspective of spatial spillover effect, this paper empirically studies the inter-industry influence mechanism, which explores the effect of excessive downstream power on upstream industry. We have proved the theoretical inference of the negative influence of buyer power on suppliers' profits and provided evidence and theoretical enlightenment in this regard, which is the unique theoretical significance of this paper and its contribution to the research field of vertical relationship.

This paper can enrich the theory of industrial organization and verify the effects and mechanisms of counterbalance power. The significance of this paper is that it not only enriches the theory of industrial organization and verifies the effects and mechanisms of countervailing power. In terms of practical significance, when facing the social problems of “high drug prices and expensive medical treatment,” it can also provide references for the anti-monopoly authorities to impose price cuts on drugs in the industrial chain, limit the price regulation and reduce the drug expenses for residences.

## 2 Conceptual framework

### 2.1 Buyer market power and pharmaceutical industry's profits

Under the context of vertical relationships in the industry chain, the role of customers has become increasingly prominent in the suppliers' decision-making process and future development prospects with the deepening division of labor and cooperation between upstream and downstream industries (2, 3). Downstream customers act as a link between suppliers' products and consumers. They transmit valuable feedback to manufacturers regarding consumer preferences, and further convey essential information related to product distribution. Moreover, they also provide sales platforms for marketing suppliers' products. Customers, as direct downstream participants with a direct interest in suppliers, are the source of suppliers' profits and the basis of their survival, as well as the most important stakeholders of the suppliers, other than the investors. The buyer market power formed by the bargaining power of downstream customers is related to the suppliers' bargaining position in market transactions, and their operations and roles in price negotiations, which further affects the suppliers' business performance (4, 5).

First, the competitive advantage perspective. Buyers in dominant negotiating positions in market transactions often seek to maximize their own profits by means such as replacing trading partners or taking products off the shelves (6–8). Decreasing the suppliers' operating efficiency and profitability could be achieved by diverse tools, such as extracting rent from suppliers, deferring payments to suppliers despite having sufficient funds, capturing the downstream market and thus firmly control sales channels, obtaining better trading conditions than competitors, appropriating suppliers' working capital through commercial credit financing, prolonging suppliers' business cycles, etc. (9, 10).

Second, the resource-dependence perspective. When the market concentration of downstream customers is high or when their purchases account for a relatively large proportion of the suppliers' sales, the suppliers are heavily dependent on downstream customers and the buyers have strong market power this moment (11–13). Relying on strong bargaining power, in addition to forcing the suppliers to make greater concessions on their profits through strategic behavior, buyers also puts the suppliers at risk of terminating the relationships at any time and replacing the counterparties (14, 15). Due to the practical need to prevent the loss of customer resources and the breakdown of cooperative relationships, suppliers must actively maintain close contractual relationships with downstream customers so as to ensure the stability of their market share (16, 17). Thus, suppliers need to pay a high cost to maintain these contractual relationships, while reducing their own profitability (18, 19).

Third, the perspective of relationship-specific investments and transaction costs. When there is a particular relationship-specific investment between a supplier and a buyer, the supplier' operational flexibility and the right of independent decision-making is also reduced by the increased switching cost (20–22). In case a downstream customer is facing a financial crisis or is on the verge of bankruptcy, given the stable sales channels, relatively fixed trading rules, and mutually agreed trading mechanisms established between the supplier and the downstream customer over a long period of time, the supplier is likely to face the risk of sales disruption due to the termination of the trading relationship if it is difficult to find another alternative buyer within a short period of time (23, 24). In this case, the relationship-specific assets between the supplier and the downstream customer will lose a lot of or even their entire original value (25–27), thereby pushing up the supplier's transaction costs, resulting in lower market performance.

Fourth, the perspective of operational and financial risks. When the market power of the downstream buyers is strong, it means that there is higher market concentration of the downstream customers and more frequent trading relationship between the suppliers and the downstream customers (28–30). This over-dependence on close trading relationships tends to increase the potential risks for suppliers (31–33). On the one hand, it is highly likely that downstream customers will achieve a backward integration strategy by virtue of their competitive advantage and break their partnership with the suppliers, the suppliers then face difficulty in bringing products to market, and their business risks rise (34–36). On the other hand, in case a downstream customer is facing a financial crisis or is on the verge of bankruptcy, it will largely transfer its own financial risk to its trading partners, resulting in a significant reduction in suppliers' profitability (37–39).

Hypothesis H_1_a: buyer power of local medical institutions can diminish the profits of the local pharmaceutical industry via various means of vertical control.
Hypothesis H_1_b: local medical institutions, following the same mechanism in market transactions, can also transmit the market power to pharmaceutical industries in other regions and depress their profits.
Hypothesis H_1_c: buyer power of local medical institutions reduces pharmaceutical industry profits in all provinces.

### 2.2 Buyer market power, corporate size, and profit

Large-scale enterprises are more effective innovators and have a bigger impact on the market (40). Their competitive edge comes from a variety of business models and the advantages of economies of scale, which allow them to cut costs, more effectively use resources for R&D, and spread risk. These benefits improve the external environment for innovation within these businesses, fostering their passion for creative endeavors and giving them more chances to succeed in innovation (41–43). This relieves the pricing pressure and the risk of innovative rents being extracted imposed by powerful downstream consumers to some extent, which ultimately increases their overall profitability (44–47). Based on this, we propose the following hypothesis.

H_2_: The pharmaceutical industry is more likely to offset the negative impact of the buyer market power on profits as companies grow in size.

### 2.3 Buyer market power, asset specificity, and profit

Due to its higher level of asset specialization, the pharmaceutical sector differs from the whole industry in a number of distinctive ways. Specific assets are characterized by difficult conversion toward alternative uses, low liquidity, and high conversion costs. As a result, attempts to rearrange these specific assets have a major negative impact on their value (48, 49). Suppliers are vulnerable to post-negotiation risks related to the specific asset investment when they are faced by powerful downstream purchasers. These risks include the possibility for exploitation, entrapment, and unfavorable posture (50, 51). As a result, suppliers' revenues are reduced since they must comply with the stringent requirements put out by buyers (52). Thus, we propose the following hypothesis:

H_3_: The pharmaceutical industry experiences reduced profitability when there is a higher degree of asset specificity, as greater buyer market power exerts a negative influence.

## 3 Methodology

### 3.1 Establishment of vertical relationship

Sales revenue is the main source of profit for industrial businesses, the claims presented in this research are well-supported by the substantial correlation between profit and sales revenue, which is measured at 0.9843 by using the Stata14.0 program. It is crucial to understand that the pharmaceutical industry in each particular location depends on sales revenue from 30 other buyer markets in addition to the local buyer market when taking that sector into account. A complex dynamic is introduced by the vertical connection between the 31 pharmaceutical corporations acting as sellers and the 31 medical enterprises acting as buyers.

**Figure 1:**
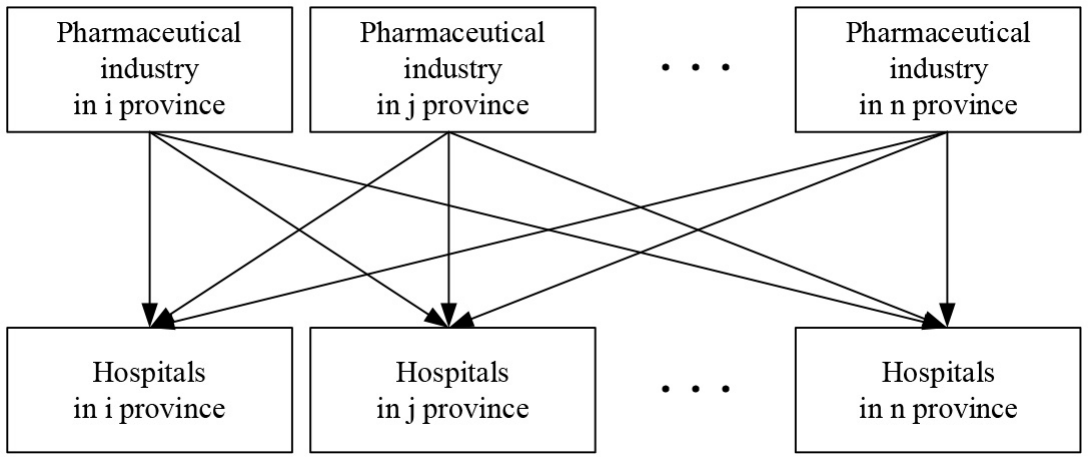
Interaction diagram of regional vertical relationship.

**Figure 2:**
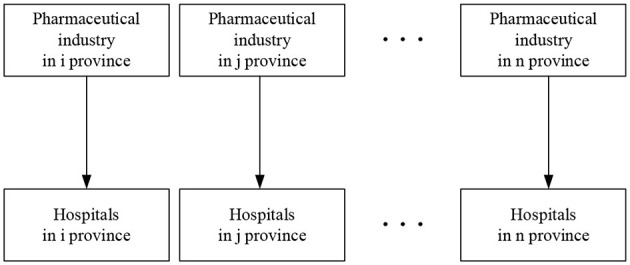
Vertical 1 × 1 relationship diagram.

In others terms, hospitals in any region have an impact on the sales income (and subsequently the profit) of both local pharmaceutical industry and pharmaceutical industries in nearby regions. Figure 1 shows a vertical spatial relationship between the independent and dependent variables, as opposed to the more obvious 1 × 1 relationship depicted in Figure 2. In essence, the independent variable has spatial and geographic implications on the dependent variable.

### 3.2 Establishment and test of spatial Durbin model (SDM)

By taking into account the spatial linkages between all of the sections, the spatial panel analysis differs from the traditional panel technique. As geographic information is added based on the differentiated individual variables, it improves the accuracy of estimation results and captures the influence of local independent variables on local and regional dependent variables by including spatial data with individual variables. Its usefulness is increased as a result.

Spatial panel models include spatial autoregressive model (SAR), spatial error model (SEM), and spatial Durbin model (SDM). The general form of the spatial panel models is as follows:


$$\begin{array}{l} {\text{y}}_{\text{i},\text{t}}=\rho \overset{\text{N}}{\underset{\text{j}=1}{\sum }}{\text{W}}_{\text{i},\text{j}}{\text{y}}_{\text{j},\text{t}}+\beta {\text{x}}_{\text{i},\text{t}}+\delta \overset{\text{N}}{\underset{\text{j}=1}{\sum }}{\text{W}}_{\text{i},\text{j}}{\text{x}}_{\text{j},\text{t}}+{\text{u}}_{\text{i}}+\eta_{\text{t}}+\varepsilon_{\text{i},\text{t}} \end{array}$$ (1)

For the SDM model, its estimation procedure is as follows. Firstly, Wald or LR statistics are used to test the two assumptions: ① $H_{0}^{1}$:δ = 0, ② $H_{0}^{\text{2}}$:δ + ρβ = 0. The two sets of hypothesis conditions are employed to determine whether it is possible to simplify the SDM into SAR or SEM. If both sets of hypotheses are disproven, the choice is in favor of the SDM. Secondly, The Hausman test is applied to determine the appropriate model specification between fixed-effect and random-effect. Thirdly, if the fixed-effect model is chosen as the appropriate specification, we need to choose among the optimal estimation model among no fixed effect, spatial fixed effect, temporal fixed effect, and spatial-temporal bidirectional fixed effect.

Compared to ordinary least squares (OLS), SDM allows decomposition of variable effects (especially the impact of market power of local medical institutions, as large buyers, on industry profits) into direct, indirect and total effects. Direct effect, indirect effect, and total effect of SDM are presented in the Appendix.

### 3.3 Sources of data, explanations of variables, and descriptive statistics

We used panel data samples from all 31 Chinese provinces, autonomous regions, and centrally managed municipalities, covering the years 2001 to 2021. The information was extracted from a number of government sources, including the China Statistics Yearbook on High Technology Industry, China Health and Family Planning Yearbook, China Industry Economy Statistical Yearbook, China Urban Life and Price Yearbook, and China Statistical Yearbook. Extreme values for all continuous variables were submitted to a Winsorization technique at the 1% level in order to reduce the influence of outliers. Stata 14.0, ArcGIS 10.2, and Matlab (56) were used for the empirical analysis and measurement. Attached Table 1 provides comprehensive information on variable units, symbols, and definitions whereas Attached Table 2 provides descriptive statistics for all variables.

**Table 1:** The regression results for inverse distance spatial weight matrix.

**Explanatory variable**	**Profit**			
	**Direct effect**	**Indirect effect**	**Total effect**	**Pool regression**
Buyer Power	−0.028^***^ (−4.545)	−0.010 (−1.3054)	−0.038^***^ (−4.791)	−0.022^***^ (−4.042)
Seller power1	0.094^***^ (3.663)	0.582^***^ (3.925)	0.676^***^ (4.441)	0.084^***^ (3.354)
Buyer Power·Seller power1	1.850^***^ (9.591)	6.835^***^ (3.682)	8.685^***^ (4.717)	1.725^***^ (8.907)
Enterprise size	6.866^***^ (3.384)	7.298^**^ (2.094)	14.164^**^ (2.155)	6.173^***^ (2.987)
Asset specificity	−142.368^*^ (−1.774)	−199.833^**^ (−2.148)	−342.201^**^ (−2.145)	−137.992^***^ (−3.627)
Barriers to entry	0.162^***^ (11.816)	−0.365^***^ (−3.310)	−0.204^*^ (−1.887)	0.169^***^ (11.812)
Government price regulation	−20.921 (−1.542)	−1.361 (−0.022)	−22.282 (−0.378)	−21.228 (−1.566)
Market demand	3.930 (1.065)	16.014 (0.552)	19.944 (0.716)	3.581 (0.914)
Per capita GDP	2.410^**^ (2.448)	−4.664 (−1.072)	−2.254 (−0.526)	2.459^**^ (2.540)
Return on assets	248.756^***^ (3.696)	52.968 (0.175)	301.724 (0.973)	250.217^***^ (3.612)
Capital density	7,462.076^***^ (3.120)	53,054.168^***^ (5.669)	60,516.244^***^ (6.694)	7,504.870^***^ (3.165)
Density of R&D personnel	71.506^***^ (3.227)	577.733^**^ (2.548)	649.239^***^ (2.924)	61.523^***^ (2.696)
New products	0.006^**^ (2.235)	0.054^**^ (2.661)	0.060^***^ (3.003)	0.005^*^ (1.766)
*W*_1_·Bmp	−0.714 (−0.954)			
*R* ^2^	0.920			
Maximum likelihood	−1,813.259			

^***^Means that it is significant at the 0.01 level.

^**^Means that it is significant at the 0.05 level.

^*^Means that it is significant at the 0.1 level.

The z value of the regression coefficient is in parentheses; The same as below. Due to the limitation of the software program on the exact number of decimal places, Bmp·Smp in the data matrix is recorded in tens of thousands. The figures in brackets are standard errors, the same treatment has been applied to Tables 2–6 and the Appendix.

**Table 2:** The regression results for economic distance spatial weight matrix.

**Explanatory variable**	**Profit**			
	**Direct effect**	**Indirect effect**	**Total effect**	**Pool regression**
Buyer power	−0.026^***^ (−3.360)	−0.006 (−0.176)	−0.031^*^ (−1.042)	−0.026^***^ (−3.490)
Seller power1	0.067^**^ (2.642)	0.035 (0.514)	0.102 (1.607)	0.068^***^ (2.730)
Buyer Power·Seller power1	1.664^***^ (8.578)	−0.271 (−0.386)	1.393^*^ (1.958)	1.665^***^ (8.800)
Enterprise size	4.141^**^ (2.157)	15.742^***^ (3.009)	19.883^***^ (2.911)	3.569^**^ (2.319)
Asset specificity	−178.337^**^ (−2.114)	−238.217^*^ (−1.8.8)	−416.554^**^ (−2.278)	−183.224^*^ (−1.859)
Barriers to entry	0.163^***^ (11.834)	−0.005 (−0.127)	0.159^***^ (4.002)	0.164^***^ (11.734)
Government price regulation	−16.423 (−1.341)	13.223 (0.694)	−3.200 (−0.417)	−16.640 (−1.338)
Market demand	5.250 (1.396)	−9.556 (−0.925)	−4.306 (−0.417)	5.170 (1.374)
Per capita GDP	4.293^***^ (4.689)	6.407^*^ (1.978)	10.700^***^ (3.496)	4.279^***^ (4.876)
Return on assets	152.162^**^ (2.245)	734.298 (1.048)	886.460 (1.235)	155.536^**^ (2.217)
Capital density	4,098.043^*^ (1.786)	91,800.137^***^ (3.526)	95,898.179^***^ (3.666)	4,329.165^*^ (1.927)
Density of R&D personnel	63.552^***^ (2.908)	109.807 (1.221)	173.359 (0.510)	63.860^***^ (2.959)
New products	0.004 (1.546)	−0.024^***^ (−2.922)	−0.020^***^ (−2.343)	0.004 (1.469)
*W*2·Bmp	−0.166 (−0.183)			
*R* ^2^	0.975			
Maximum likelihood	−1,838.134			

^***^Means that it is significant at the 0.01 level. ^**^Means that it is significant at the 0.05 level. ^*^Means that it is significant at the 0.1 level.

#### 3.3.1 Dependent and independent variables

(1) Dependent variable: it represents the yearly net earnings of the regional pharmaceutical industry, adjusted by the GDP deflator to eliminate the influence of inflation.
(2) Independent variable: the buyer market power is the main independent variable being considered. In a novel vertical relationship where the buyer retains dominance, the goal of this study is to empirically investigate how the buyer market power affects the profitability of suppliers. Buyer market power is evaluated by counting all the hospitals in a particular area. However, enterprise size may have an impact on the indication of the seller countervailing power, measured by the total number of pharmaceutical companies in a province. Given the accessibility of data, it is possible to improve the model by including such metrics, the total assets of pharmaceutical industry in a province and the total industrial output value of the pharmaceutical industry in a province, adjusted for inflation, to assure the stability of the predictions.

In the study of the vertical relationship of industrial organization, the market power of buyers and sellers is interdependent, as the buyer market power gradually increases, the seller power plays a countering function. This is known as the interaction term of the buyer power and the seller countervailing power. As a result, the seller power functions as a countervailing power in the interaction term and acts as a moderating factor to influence the relationship between the buyer market power and the profitability of the pharmaceutical industry.

#### 3.3.2 Moderating variables and mediating variables

Enterprise size and asset specificity, two moderating factors that can be evaluated separately, are both included in this study. Asset specificity can be assessed by calculating the ratio of fixed asset investment in the pharmaceutical industry relative to the total asset investment at the end of the year, while corporate size can be calculated by dividing the total industrial output value of the pharmaceutical sector by the number of pharmaceutical companies. Additionally, we included mediating variables to investigate how the buyer market power affects supplier's profit. The pharmaceutical industry's R&D investment is a proxy for these mediating factors, and it is quantified as the ratio of internal R&D spending to sales revenue.

#### 3.3.3 Control variables

We also chose to evaluate a number of significant aspects in light of the results of the available research results. The barrier to entry is one of them, and its impact is determined by the amount of fixed asset investment. Additionally, it takes into account government price regulation, which is established by ex-factory price index of pharmaceutical industry divided by ex-factory price index of the general industrial products in a province. The difference between the current sales revenue and the previous sales revenue divided by the current sales revenue is used to compute the market demand growth rate. Per capita GDP; Return on assets (calculated as net profits divided by total assets); Capital density (determined by dividing fixed asset investments by the number of employees); the density of R&D personnel (determined by the ratio of R&D personnel to all employees), and the quantity of new product development projects are additional factors considered. The profitability of the pharmaceutical sector is significantly influenced by these control variables.

### 3.4 Estimation strategy

#### 3.4.1 Estimation method

Through this research, we tried to handle the complex longitudinal many-to-many interaction by using the SDM approach in cases when there is a paucity of granular data. Using the ordinary least squares (OLS) method could result in biased estimation findings due to the endogeneity problem, which is caused by the dependent variable being encapsulated within the independent variable. We propose using the Maximum Likelihood Estimation (MLE) method to estimate the model parameters in accordance with earlier works, including Elhorst (53) and Lee and Yu (54).

#### 3.4.2 The setting of benchmark model

The following spatial measurement model is constructed in accordance with the chosen dependent variables, independent variables, and control variables, as well as the goals of this research:


$$\begin{array}{l} Profit_{i,t}=\rho \overset{N}{\underset{j=1}{\sum }}W_{i,j}Profit_{j,t}++\beta_{1}Bmp_{j,t}+\beta_{2}Smp_{i,t}+\beta_{3}Bmp_{i,t}\\ \;\cdot Smp_{i,t}+\delta \overset{N}{\underset{j=1}{\sum }}W_{i,j}Bmp_{j,t}+\alpha X_{i,t}^{\prime }+u_{i}+\eta_{t}+\varepsilon_{i,t} \end{array}$$ (2)

In the model, *Profit*_*i, t*_ stands for the profit, *Bmp*_*i, t*_ for the buyer power, *Smp*_*i, t*_ for the seller power, ${X^{\prime }}_{i,t}$ for the matrix composed of control variables, α for the coefficient vector corresponding to the control variable matrix, β_1_ ~ β_3_ for the coefficient of the corresponding independent variable, and *W*_*i, j*_*Bmp*_*j, t*_ for the spatial spillover effect of the buyer power.

#### 3.4.3 Construction of inverse distance space weight matrix (*W*1) and economic distance spatial weight matrix (*W*2)

*W*1 is constructed based on the linear Euclidean distance between the two capital cities. When the buyer power in regions *i* and *j* is equal, the influence on area *k* diminishes as the distance between the two regions rises. This is the economic implication of the inverse distance spatial weight matrix *W*1. This idea is consistent with Tobel's first law of geography, according to which everything is connected to everything else, although connections between distant objects are weaker than connections between objects that are near to one another.

$W1=\{\begin{array}{cc} 0 & \left(i=j\right)\\ 1/d_{ij} & \left(i\neq j\right) \end{array}$, *d* is the linear Euclidean distance between the two capital cities.

When establishing the spatial weight matrix, it is essential to consider two key factors: geographical distance and economic distance. The buyer market power, serving as the central variable, plays a pivotal role in the spatial spillover effect. To put it differently, when regions *i* and *j* have the same geographical distance as region *k*, the level of impact is positively associated with the buyer power in regions *i* and *j*. In typical circumstances, when the buyer power in region i surpasses that in region j but is farther away from the seller, determining the extent of impact on region *k* becomes challenging. In accordance with previous research by Li et al. (55), an economic distance spatial weight matrix *W2* is established, incorporating both the buyer power and geographical distance factors, as illustrated below:


$$\begin{array}{l} W2=W1\cdot U=W1\cdot \mathrm{diag}\left(\frac{{\bar{E}}_{1}}{\bar{E}},\frac{{\bar{E}}_{2}}{\bar{E}},\cdots ,\frac{{\bar{E}}_{n}}{\bar{E}}\right)\\ \;U=\mathrm{diag}\left(\frac{{\bar{E}}_{1}}{\bar{E}},\frac{{\bar{E}}_{2}}{\bar{E}},\cdots ,\frac{{\bar{E}}_{n}}{\bar{E}}\right)\\ \;{\bar{E}}_{i}=\frac{1}{t_{1}-t_{0}+1}\sum_{t=t_{0}}^{t_{1}}E_{i,t}\\ \;\bar{E}=\frac{1}{N\left(t_{1}-t_{0}+1\right)}\sum_{i=1}^{N}\sum_{i=t_{0}}^{t_{1}}E_{i,t} \end{array}$$

In the equation, *U* stands for the diagonal matrix, which is used to measure the relative size of the buyer power in different regions; $\overset{-}{E_{i}}$ stands for the average of the total number of hospitals in *i* region, ${\bar{E}}_{i}$ is the average of the total number of hospitals in all provinces, autonomous regions, and municipalities directly under the central government, and *t* for different periods, *t* ∈ [2001, 2021], *t*_0_ = 2001, *t*_*l*_ = 2021. *W*2 stands for the influencing mechanism of the buyer under the joint action of market power and spatial location on the profit of pharmaceutical industry in different regions.

### 3.5 Robustness test

#### 3.5.1 Cluster standard error regressions for the OLS

In the further test, the basic econometric specification is also added to this paper, we use cluster standard error regressions for the OLS as one of the robustness of the estimates, the regression results are shown in Attached Table 3.

Table 3Regression results for heterogeneous competitive environment.

**Table:** 

**Panel A classification by geographical region**						
	**Eastern regions**		**Central regions**		**Western regions**	
**Buyer power**	*W*1	*W*2	*W*1	*W*2	*W*1	*W*2
Direct effect	−0.266^*^ (−1.834)	−0.397^*^ (−1.827)	−0.186^*^ (−1.797)	−0.448^*^ (−1.801)	−0.586^***^ (−3.004)	−0.608^***^ (−4.742)
Indirect effect	−0.098 (−1.297)	−0.063 (−1.278)	−0.042 (−0.897)	−0.112 (−1.416)	−0.188 (−1.137)	−0.315 (−1.304)
Total effect	−0.364^*^ (−1.776)	−0.460^*^ (−1.815)	−0.231^*^ (−1.860)	−0.560^*^ (−1.791)	−0.773^***^ (−3.118)	−0.924^***^ (−3.303)
Pool regression	−0.220^*^ (−1.858)	−0.325^*^ (−1.879)	−0.191^*^ (−1.850)	−0.404^*^ (−1.862)	−0.524^***^ (−3.920)	−0.584^***^ (−3.178)
Control Variables	Control	Control	Control	Control	Control	Control

**Table:** 

**Panel B classification by capital intensity**				
	**High capital intensity**		**Low capital intensity**	
**Buyer power**	*W*1	*W*2	*W*1	*W*2
Direct effect	−0.125^*^ (−1.826)	−0.255^*^ (−1.822)	−0.375^***^ (−5.008)	−0.662^***^ (−3.381)
Indirect effect	−0.044 (−0.917)	−0.090 (1.070)	−0.092 (−1.271)	−0.129 (−1.267)
Total effect	−0.169^*^ (−1.832)	−0.345^*^ (−1.903)	−0.466^***^ (−2.992)	−0.791^***^ (−4.098)
Pool regression	−0.113^*^ (−1.777)	−0.244^*^ (−1.791)	−0.399^***^ (−4.579)	−0.689^***^ (−3.639)
Control variables	Control	Control	Control	Control

^***^Means that it is significant at the 0.01 level. ^**^Means that it is significant at the 0.05 level. ^*^Means that it is significant at the 0.1 level.

#### 3.5.2 Measurement of the seller power according to the total assets of the regional pharmaceutical industry and total industrial output value of the regional pharmaceutical industry

**Figure 3:**
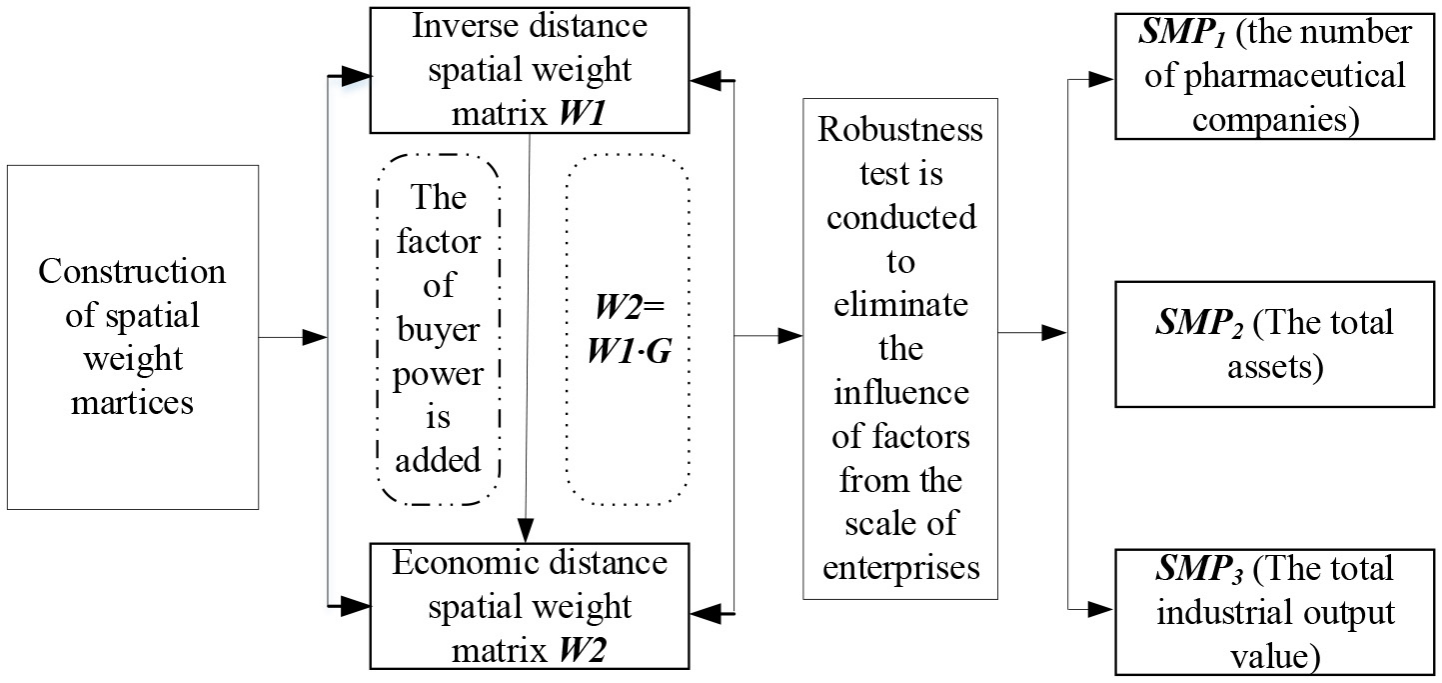
Robustness test of the sensitivity of results.

It becomes vital to carry out a robustness test when comparing the seller countervailing power in region *i* relative to region j by taking the number of pharmaceutical businesses into consideration. This criteria is crucial to removing the influence of the scale of enterprises from the calculation of relative power. To be clear, even if both locations have an equal number of pharmaceutical businesses, their relative market power may vary if the pharmaceutical industry in one is more developed and includes larger organizations. Therefore, it is essential to use measurement indicators from diverse viewpoints. Examining the total assets of the regional pharmaceutical industry and the total industrial output value of the regional pharmaceutical industry is one way to address this problem. We use Figure 3 to assess the sensitivity of results to different measurement and spatial weight matrices, it is presented as follows.

## 4 Results

### 4.1 Spatial weights matrices results

#### 4.1.1 Regression analysis for inverse distance space weight matrix (*W*1)

After testing, with respect to inverse distance space weight matrix *W*1, all of the Wald statistics reject the null hypothesis of δ = 0 and δ + ρβ = 0 at the significance level of 0.01, indicating that the spatial Durbin model is the best choice. Hausman test determines that the spatial-temporal bidirectional fixed effect is the optimal estimation model, and the regression results are shown in Table 1.

**Figure 4:**
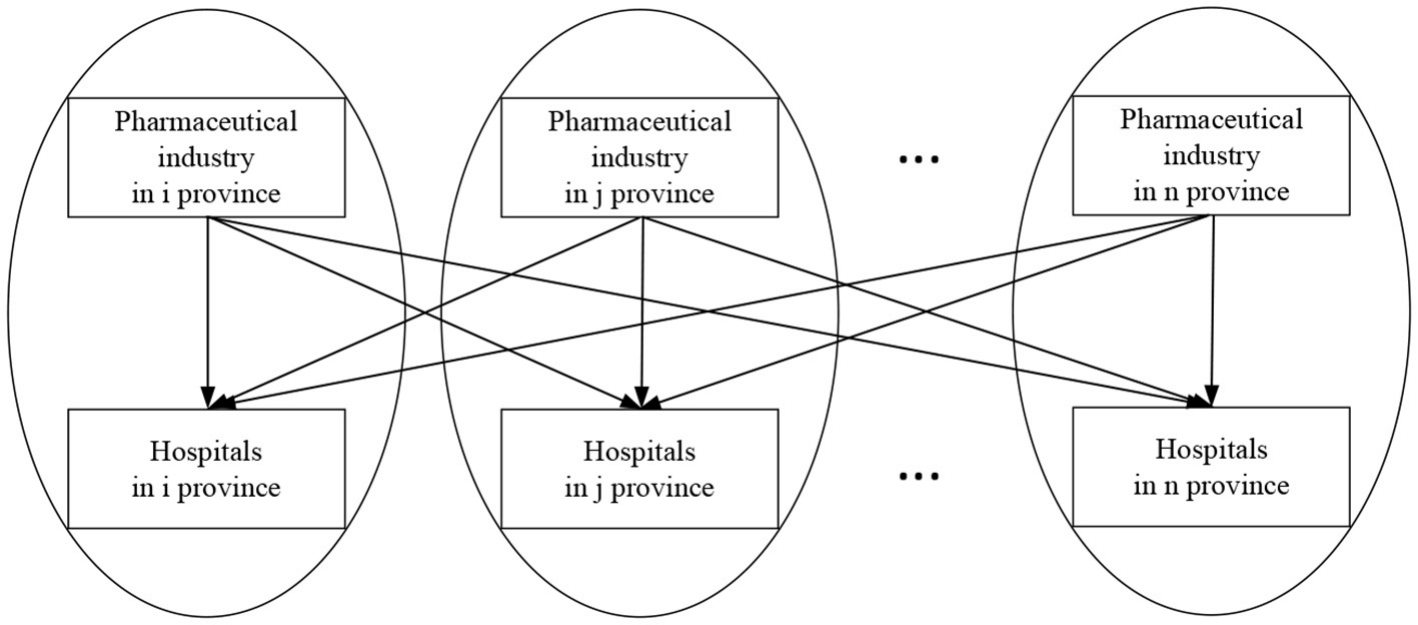
Economic implications for vertical markets.

From Table 1, it can be known that:

(1) The coefficient δ of spatial spillover effect of the core variable, buyer market power, exhibits a negative direction, and it does not demonstrate statistical significance, indicating that the spatial spillover effect of buyer market power is not significant.
(2) The buyer market power has a noteworthy negative direct effect with a significance level of 0.01. This suggests that the buyer market power of local medical institutions can substantially diminish the local pharmaceutical industry's profits, confirming Hypothesis H_1_a. Furthermore, the adverse indirect effect implies that local medical institutions, while decreasing the local pharmaceutical industry's profitability through diverse vertical strategic actions, can also employ this same mechanism to transfer their market power to pharmaceutical industries in other regions, thereby reducing their profits as well. Therefore, Hypothesis H_1_b is verified. Nonetheless, the insignificance of the coefficient suggests that within the context of spatial spillover effects in the transmission mechanism, the local buyer market power does not have a significant impact on reducing pharmaceutical industry's profits in other regions. This aligns with the observed direction and significance level of *W*1_*ij*_·*Bmp*_*jt*_. The overall effect is negative, indicating that it reduces the profit of the pharmaceutical industry in all regions. Therefore, Hypothesis H_1_c is verified.
(3) The absolute value of the coefficient of indirect effect is significantly lower than the absolute value of the coefficient of direct effect. This suggests that market players within the same region have closer economic ties and market transaction relationships. In this situation, the pharmaceutical industry, acting as the supplier, is subject to a stronger vertical constraint from the medical industry, acting as the buyer. Additionally, rather than extending to other regions, the main sales channel for medications primarily operates inside the local area. This circumstance creates what can be called an “economic circle.” Figure 4 provides a visual representation of this idea, showing how the downstream hospitals in region *i* and the upstream pharmaceutical sector are essentially encircled in this economic circle.
(4) The coefficient of direct effect of *Bmp*_*it*_·*Smp*_1*it*_showing the interaction of the vertical market power is positive and significant at the level of 0.010. According to Equation 2, the partial derivation of the dependent variable of profit to the independent variable of the buyer power can lead to the expression: ∂*Profit*_*it*_/∂*Bmp*_*it*_ = β_1_ + β_3_*Smp*_*it*_. In which, Q25(Smp) = 384, median(Smp) = 607, mean(Smp) = 684.5685, Q75 (Smp) = 926.5. In this way, the effect of profit as a function of Bmp depends on the value of Smp is well reflected, which avoids the effect of Bmp would be the effect of this variable when Smp = 0. If the seller countervailing power is taken into account as a moderating element, it can help balance the negative impacts of the buyer power on the seller's profitability, thus lessening the negative effects of the buyer power. In essence, pharmaceutical companies have some market power, namely, if they have a countervailing power, they may be able to increase their own profits. The significance of coefficient suggests that both buyers and sellers in the same region exist as market trading entities with closer economic relationships, and the downstream industry has a larger vertical influence over suppliers. This is consistent with the fact that the absolute value of buyer power has a much higher direct effect than it does an indirect one.
(5) As to control variables, barrier to entry reduce industry competition, as a result, incumbent businesses can profit for a longer period of time from being the first to enter the market. Pharmaceutical companies may be forced to abandon R&D initiatives with uncertain or low-profit prospects due to government-mandated price reductions and maximum price regulations for particular medications. Expected returns in the pharmaceutical industry are consequently drastically reduced. Strong market demand produces a steady and regular cash flow, which boosts the profits made by the pharmaceutical industry. Profitability in the pharmaceutical industry is positively correlated with the degree of economic development in a certain area. Investments in innovation are supported by revenue from business growth and profitability, which also improves business performance. Employee training and advanced knowledge are linked to a company's capacity for research and development, which considerably boosts earnings. The pharmaceutical industry has a high concentration of R&D workers, which is indicative of the industry's overall R&D capacity and long-term growth potential, which benefits performance. Market demand is determined by a drug's efficacy, with specialized pharmaceuticals with intricate production methods and high levels of science and technology displaying promising market potential and sizable earnings in comparison to conventional drugs. National patent protection technology also enables brand-name medicine producers to continue profiting from their patents for an extended length of time.

#### 4.1.2 Regression analysis for economic distance spatial weight matrix (*W*2)

After testing, the Wald statistics of *W*2 reject the null hypothesis of δ = 0 and δ + ρβ = 0 at the significance level of 0.01 both, which means that the SDM is the best choice. The Hausman test indicates that the spatial fixed effect is the optimal estimation model, and the regression results are shown in Table 2.

*W*2 further contends that buyer power has a negative impact on local and other regional earnings of pharmaceutical industry. However, there are no noticeable knock-on effects in other places as a result of this impact. Our hypotheses H1a, H1b, and H1c are therefore supported. There are closer trading relationships and economic connections between the vertical industry chain market entities in the same region. When pharmaceutical industry has a countervailing power, they may be able to increase their own profits. In terms of direction and significance level, the coefficients of the control variables closely match the results shown in Table 1.

### 4.2 Robustness test results

#### 4.2.1 Regression analysis for OLS

As can be seen in attached Table 3, according to the coefficient symbol and significance level of core variables *Bmp*_*i, t*_, *Smp*_*i, t*_, *Bmp*_*it*_·*Smp*_2*it*_ and control variables, the robustness of the regression results can also be proved When the total number of regional hospitals is used to measure the buyer power, and the number of pharmaceutical enterprises is used to measure the seller power under the setting of the inverse distance spatial weight matrix and the economic distance spatial weight matrix.

#### 4.2.2 Regression analysis for measurement of the seller power according to the total assets of the regional pharmaceutical industry and total industrial output value of the regional pharmaceutical industry

When buyer power is gauged by assessing the combined count of regional hospitals, while the total assets of the regional pharmaceutical industry are used to measure the seller countervailing power. According to the Hausman test, the results of the robustness test *W*1 using time fixed effects are shown in Panel A of attached Table 4, whereas the results of the robustness test *W*2 using both space and time bidirectional fixed effects are shown in Panel B of attached Table 4. It also proves the robustness of the regression results under the setting of *W*1 and *W*2.

**Table 4:** Regression results of corporate size as a moderating variable.

**Explanatory variable**	**Profit**			
	**Direct effect**	**Indirect effect**	**Total effect**	**Pool regression**
**Panel A** *W*1				
Buyer power	−0.369^**^ (−2.157)	−0.109 (−0.917)	−0.478^**^ (−2.453)	−0.341^**^ (−2.216)
Enterprise size	5.597^***^ (4.679)	10.121^*^ (1.886)	15.718^*^ (1.816)	5.179^***^ (3.619)
Buyer power·Enterprise size	3.641^***^ (2.774)	9.168^**^ (2.126)	12.809^**^ (2.371)	3.215^***^ (3.091)
Control variables	Control	Control	Control	Control
**Panel B** *W*2				
Buyer power	−0.706^**^ (−2.422)	−0.243 (−0.752)	−0.949^**^ (−2.355)	−0.749^**^ (−2.356)
Enterprise size	3.546^*^ (1.860)	22.175 (1.507)	25.721^**^ (2.337)	3.383^**^ (2.158)
Buyer power·Enterprise size	4.091^***^ (3.561)	5.658 (1.121)	9.750^**^ (2.189)	4.233^***^ (3.237)
Control variables	Control	Control	Control	Control

^***^Means that it is significant at the 0.01 level. ^**^Means that it is significant at the 0.05 level. ^*^Means that it is significant at the 0.1 level.

The buyer power is gauged by assessing the combined count of regional hospitals, while seller countervailing power is evaluated by considering pharmaceutical industry's total industrial output. In accordance with the Hausman test, the outcomes of the robustness tests, labeled as *W*1 and conducted with space and time bidirectional fixed effects, are displayed in Panel A of attached Table 5. Additionally, Panel B of attached Table 5 presents the findings of the *W*2 robustness test, which employs a time fixed effect. The robustness of the regression findings are further demonstrated.

**Table 5:** Regression results of asset specificity as a moderating variable.

**Explanatory variable**	**Profit**			
	**Direct effect**	**Indirect effect**	**Total effect**	**Pool regression**
**Panel A** *W*1				
Buyer power	−0.247^**^ (−2.171)	−0.057 (−1.326)	−0.303^*^ (−1.856)	−0.212^**^ (−2.324)
Asset specificity	−165.058^*^ (−1.871)	−217.116^**^ (−2.306)	−382.174^**^ (−2.164)	−170.153^*^ (−1.796)
Buyer power·Asset specificity	−0.241^***^ (−3.030)	−0.512^**^ (−2.116)	−0.753^**^ (−2.216)	−0.272^***^ (−3.647)
Control variables	Control	Control	Control	Control
***Panel B*** *W*2				
Buyer power	−0.425^***^ (−2.997)	−0.116 (−1.171)	−0.541^**^ (−2.143)	−0.463^***^ (−3.371)
Asset specificity	−189.784^*^ (−1.907)	−252.656^**^ (−2.613)	−442.441^*^ (−1.863)	−195.271^**^ (−2.124)
Buyer power·Asset specificity	−0.512^*^ (−1.872)	−0.687 (−0.912)	−1.199^**^ (−2.448)	−0.501^**^ (−2.034)
Control Variables	Control	Control	Control	Control

^***^Means that it is significant at the 0.01 level. ^**^Means that it is significant at the 0.05 level. ^*^Means that it is significant at the 0.1 level.

### 4.3 Further analysis of heterogeneous competitive environments

From the perspective of vertical relationships in the industrial chain, the supplier's competitive position and relative advantage inside the vertical market are significantly shaped by the heterogeneous external competitive environment. As a result, this further affects the supplier's negotiating position when dealing with buyers, which ultimately affects their innovation performances. In this study we further groups the pharmaceutical industry according to different categories, and analyzed heterogeneity in terms of geographic location and capital intensity. Table 3 presents the results of our regression analysis.

In the situation where data is unavailable, this paper can only obtain data on the number of new products developed. We use the mean value of capital intensity of the pharmaceutical industry as categorical variables, the pharmaceutical industry is divided into different categories of high capital density and low capital density. By analyzing the correlation between capital intensity in the pharmaceutical industry and the number of new products developed using Stata 14.0 program, it can be concluded that the correlation coefficient between the two variables is 0.9194. The result indicates that capital intensity has a significant positive correlation with the number of new products developed.

In the further analysis combined with Table 3 above, with respect to inverse distance space weight matrix *W*1, the coefficients of indirect effect do not pass the significance test, in accordance with the regression results in Panels A and B of Table 3, which are consistent with the influence and transmission mechanism of local buyer market power on pharmaceutical industry's profit in other regions as shown in Tables 1, 2. The coefficient of *Bmp*_*it*_ of pharmaceutical industry located in western region (defined by low capital intensity) shows extremely significant coefficients of direct and total effect at the 0.01 level. The pharmaceutical industry located in the eastern and central regions (characterized by high capital intensity) are weakly significant. The finding is evidence buyer power is not of great concern for, at least, value-adding innovations of new products developed, or even potential patented value-adding innovations. Additionally, the absolute values of the coefficients of *Bmp*_*it*_ for the direct effect, indirect effect, and total effect for the pharmaceutical industry in the western region (defined by low capital intensity) are noticeably higher than those for the pharmaceutical industry in the eastern and central regions (characterized by high capital intensity). This pattern is valid under the *W*2 conditions. An increased reliance on sellers in market transactions has resulted from the growth of pharmaceutical technology in the eastern and central regions as well as pharmaceutical businesses' ability in research and development to produce new pharmaceuticals. As a result, this has limited the outside options that downstream customers have, increasing the supplier's bargaining power. High capital intensity businesses are more likely to seek innovation, and their capacity to create innovative products strengthens their clout in vertical transactions. In consequence, this reduces the downstream customers' market power while increasing the suppliers' countervailing power.

### 4.4 The test of moderating effect

In order to test Hypothesis 2 and Hypothesis 3, we established Model (3):


$$\begin{array}{l} \begin{array}{l} {Profit}_{i,t}=\rho \overset{N}{\underset{j=1}{\sum }}W_{i,j}Ip_{j,t}+\beta_{1}{Bmp}_{i,t}+\beta_{2}K_{i,t}+\beta_{3}{Bmp}_{i,t}\cdot K_{i,t}\\ \;+\delta \overset{N}{\underset{j=1}{\sum }}W_{i,j}{Bmp}_{j,t}+\alpha_{1}{Govr}_{i,t}+\alpha_{2}{Demand}_{i,t}\\ \;+\alpha_{3}{Reg}_{i,t}+\alpha_{4}{Sprofit}_{i,t}+\alpha_{5}{Roa}_{i,t}+\alpha_{6}{Capital}_{i,t}\\ \;+u_{i}+\eta_{t}+\varepsilon_{i,t} \end{array} \end{array}$$ (3)

In the model, the moderating variable *K*_*i, t*_ includes the two variables of corporate size (Size) and asset specificity (Asset). The regression results are shown in Tables 4, 5.

Through the partial derivation of the dependent variable innovation performance to *Bmp*_*it*_ in Equation 3, the following equation can be obtained: ∂*Profit*_*it*_/∂*Bmp*_*it*_ = β_1_ + β_3_*Size*_*it*_; combined with the coefficient signs and significance levels of *Bmp*_*it*_, *Size*_*it*_, *Bmp*_*it*_·*Size*_*it*_ as shown in Table 4, the following conclusion can be reached: when corporate size is used as a moderating element, it can improve the vertical squeeze of downstream customer market power on the profits of the pharmaceutical industry, and weaken the negative effect of buyer power. In other words, the scale of the enterprise can play a positive regulatory role, to a certain extent, alleviate the adverse effects of buyer power. As a result, Hypothesis H_2_ is confirmed.

Through the partial derivation of the dependent variable innovation performance to *Bmp*_*it*_ in Equation 3, the following equation can be obtained: ∂*Profit*_*it*_/∂*Bmp*_*it*_ = β_1_ + β_3_*Asset*_*it*_. Combined with the coefficient signs and significance levels of *Bmp*_*it*_, *Asset*_*it*_, *Bmp*_*it*_·*Asset*_*it*_ in Table 5, the following conclusion can be reached: when asset specificity is used as a moderating factor, it has the ability to intensify the damaging impact of downstream customers' market dominance on pharmaceutical industry's earnings, and enhance the negative effects of the buyer market power. To put it another way, asset specificity can work as a negative moderating element within the pharmaceutical industry, further aggravating the unfavorable influence of the buyer market power. Consequently, this validates the validity of Hypothesis H_3_.

### 4.5 Test of action mechanism

The pharmaceutical industry's cutting-edge technological features attract R&D spending, which is essential for boosting product competitiveness, growing market presence, and attaining steady growth. Downstream buyers using their market power to limit suppliers' R&D spending will inevitably lead to insufficient innovation spending, which will stifle performance in this area. In this study, we aim to strengthen the logical framework by studying the impact mechanism utilizing mediating variables given that the inverse association between a buyer's influence and innovation performance in the pharmaceutical industry has already been verified. Model (4) is introduced in this study to examine if buyer market power affects the pharmaceutical industry's R&D investment.


$$\begin{array}{l} \begin{array}{c} {Rnd}_{i,t}=\rho \overset{N}{\underset{j=1}{\sum }}W_{i,j}{Rnd}_{j,t}+\beta_{1}{\text{Bmp}}_{i,t}+\beta_{2}{Smp}_{i,t}+\beta_{3}{Bmp}_{i,t}\\ .{Smp}_{i,t}+\delta \overset{N}{\underset{j=1}{\sum }}W_{i,j}{Bmp}_{j,t}+\alpha X_{i,t}+u_{i}+\eta_{t}+\varepsilon_{i,t} \end{array} \end{array}$$ (4)

In the model, *Rnd*_*i, t*_ stands for R&D investment and the remaining variables have the same meanings as those in Equation 2. It is worth mentioning that in the pharmaceutical industry within any given region, when R&D investment is measured as the ratio of internal R&D spending to sales revenue, considering that the R&D spending is conducted across the entire country and the sales revenue comes from both local and other regional buyer markets, there exists a complex interaction in the vertical relationship. This interaction remains to be addressed through a spatial measurement model. The specific regression results are presented in Table 6.

**Table 6:** Buyer power and R&D investment in the pharmaceutical industry.

**Independent variable**	**Rnd**			
	**Direct effect**	**Indirect effect**	**Total effect**	**Pool regression**
**Panel A** *W*1				
*Buyer power*	−0.132^**^ (−2.056)	−0.031 (−1.124)	−0.163 (−1.350)	−0.131^*^ (−1.957)
Control variables	Control	Control	Control	Control
**Panel B** *W*2				
Buyer power	−0.422^***^ (−2.952)	−0.031 (−0.821)	−0.453^***^ (−3.194)	−0.408^**^ (−2.338)
Control variables	Control	Control	Control	Control

Due to the limitation of the software program on the exact number of decimal places, Bmp, Smp, and Bmp·Smp in the data matrix are all recorded in tens of thousands. ^***^Means that it is significant at the 0.01 level. ^**^Means that it is significant at the 0.05 level. ^*^Means that it is significant at the 0.1 level.

Table 6 shows that the coefficient of *Bmp*_*it*_ demonstrates substantial negative values at varied levels in the regression findings for *W*1 and *W*2. This suggests that the influence of buyer power causes upstream suppliers in the industry chain to reduce their R&D investments, which has a negative impact on earnings owing to innovation limits. This thereby validates the method by which buyer power affects the earnings of the pharmaceutical industry.

In further research, this paper explores the subdivision of R&D spending into product R&D spending and process R&D spending in depth, so as to observe the impact of buyer power on the two forms of R&D spending respectively. *Rnd*_*i, t*_ is divided into *Product* *Rnd*_*i, t*_ and *ProcessRnd*_*i, t*_, the remaining variables have the same meanings as those in Equation 2. The specific regression results are presented in Tables 7, 8 respectively.

**Table 7:** Buyer power and product R&D investment in the pharmaceutical industry.

**Independent variable**	**Product Rnd**			
	**Direct effect**	**Indirect effect**	**Total effect**	**Pool regression**
**Panel A** *W*1				
Buyer power	−0.078^**^ (−2.371)	−0.019 (−1.116)	−0.094 (−1.437)	−0.066^**^ (−2.099)
Control variables	Control	Control	Control	Control
**Panel B** *W*2				
Buyer power	−0.257^***^ (−3.855)	−0.017 (−0.659)	−0.267^***^ (−4.337)	−0.209^**^ (−2.616)
Control variables	Control	Control	Control	Control

Due to the limitation of the software program on the exact number of decimal places, Bmp, Smp, and Bmp·Smp in the data matrix are all recorded in tens of thousands. ^***^Means that it is significant at the 0.01 level. ^**^Means that it is significant at the 0.05 level. ^*^Means that it is significant at the 0.1 level.

**Table 8:** Buyer power and process R&D investment in the pharmaceutical industry.

**Independent variable**	**Process Rnd**			
	**Direct effect**	**Indirect effect**	**Total effect**	**Pool regression**
**Panel A** *W*1				
Buyer power	−0.054^**^ (−2.368)	−0.014 (−1.385)	−0.079 (−1.447)	−0.049^*^ (−1.896)
Control Variables	Control	Control	Control	Control
**Panel B** *W*2				
Buyer power	−0.165^***^ (−3.649)	−0.014 (−0.968)	−0.179^***^ (−3.822)	−0.178^**^ (−2.035)
Control variables	Control	Control	Control	Control

Due to the limitation of the software program on the exact number of decimal places, Bmp, Smp, and Bmp·Smp in the data matrix are all recorded in tens of thousands. ^***^Means that it is significant at the 0.01 level. ^**^Means that it is significant at the 0.05 level. ^*^Means that it is significant at the 0.1 level.

According to Tables 7, 8, the coefficient of *Bmp*_*it*_ demonstrates substantial negative values at varied levels in the regression findings for *W*1 and *W*2. This suggests that the influence of buyer power causes upstream suppliers in the industry chain to reduce their product R&D investment and Process R&D Investment both, the negative effect on earnings still holds when different forms of innovative activities are constrained.

## 5 Conclusions and policy recommendations

### 5.1 Conclusions

Using the data of China's pharmaceutical industry and medical industry from 2001 to 2021, this paper selects the pharmaceutical industry, a typical industry with prominent buyer power, for research based on the realistic background of China's market. This paper studied the upstream and downstream rivalries in the pharmaceutical industry in the provinces of China. From the perspective of spatial spillover effect, we construct a vertical relationship between pharmaceutical industries in the 31 provinces of the seller and medical industries in the 31 provinces of the buyer, and empirically studies the influence mechanism between industries, which explores the influence of market power from downstream customers on the profits of pharmaceutical industry in industrial organizations, as well as the effect on upstream industries when the downstream power is too powerful. We found the following conclusions.

(1) The buyer power faced by pharmaceutical industry (the large buyer counterbalance power developing to a certain stage) does exist and it is universal. When the position of the buyer and the seller changes, in the case of the new buyer-led vertical relationship, the distortion of the vertical relationship caused by buyer power will have a negative impact on the upstream industry. This effect is in contrast to the beneficial effect of buyer counterbalance power (1).
(2) Factors that affect the decision-making of enterprises include not only the structural characteristics of the industry in the horizontal market, but also the vertical power characteristics in the industrial organization. Downstream industry characteristics also play a role in the decision-making of upstream industries, in which market power is the typical representative. This paper extends the industrial chain hierarchy, the market power from downstream customers in the industrial organization is included in the analysis of the impact on the profits of upstream industry. The results show that the local buyer power reduces the profit of the pharmaceutical industry in the local and other provinces at the same time, but the spatial spillover effect is not significant.
(3) The absolute value of the coefficient of direct effect is significantly greater than that of the indirect effect. The pharmaceutical industry and medical institutions within the same region have closer market transaction relationship and economic connection. In this situation, the pharmaceutical industry, acting as the supplier, is subject to a stronger vertical constraint from the medical industry, acting as the buyer.
(4) The interaction term of market power indicates that when the seller counterbalance power is taken as the moderating variable, the counterbalance power of the supplier can improve the negative influence of the buyer power on its own profit and weaken the negative effect of buyer power. In other words, the pharmaceutical industry also has market power, when it has a counterbalance power, its own profits would increase.
(5) When we take the number of regional hospitals as the buyer power, the total assets and the total industrial output value of the pharmaceutical industry as the measure of the seller power, the conclusions above are also proved to be robust.
(6) This article explores the differentiation of countervailing power among different categories of pharmaceutical industries in a heterogeneous competitive environment, and confirms that the pharmaceutical industry in the eastern and central regions, as well as the high capital density (western regions, low capital density), have stronger (weaker) countervailing power compared to buyers.
(7) This article confirms that enterprise size can alleviate the adverse impact of buyer market power on the profits of the pharmaceutical industry, while asset specificity can exacerbate this adverse impact. Furthermore, this study explores its mechanism of action and confirms that the underlying mechanism behind the negative impact of buyer market power on the profits of the pharmaceutical industry is to reduce the R&D investment level of the latter.

### 5.2 Policy recommendations

The pharmaceutical industry chain consists of the Active Pharmaceutical Ingredient suppliers (API suppliers), the pharmaceutical industry, and the medical industry (mainly hospitals as the distribution terminal). The profit shares of each stakeholder in the vertical market come from patients as a specific consumer group. This study empirically concludes that the market power of medical institutions drives the profits of the pharmaceutical industry to flow to medical institutions and reduces the performance of the pharmaceutical industry. The immediate policies are as follows: (1) Regulatory bodies should cater for heterogenous preferences of customers to encourage the development of private hospitals, those who prefer (lower-priced) public hospitals will use them and those who prefer (higher priced) private facilities get what they want, thus reducing society's reliance on public hospitals. Increasing the number and geographical distribution of community pharmacies in an equitable fashion so as to increase market demand facing manufacturers and extend patients' choices of medicines, in order to increase access to care (demand) for patients. In addition to overseeing the wholesale price of medicines, regulators should also focus on supervising the price of medicines sold at medical terminals. (2) Regulatory bodies should strictly regulate and control the transaction behavior of downstream customers, especially the medical industry, and crack down on all kinds of unfair competition imposed on pharmaceutical companies by the medical industry drawing on their dominant market position and abusing their market power. (3) Pharmaceutical companies in weaker market positions or complex competitive environments need to continue harmonious relationships with customers while also holding high cash reserves to guard against unfavorably effect on short-term production and operations brought by cash-flow risks and adverse external shocks.

The pharmaceutical industry in China faces powerful downstream buyers, and under the context that “hospitals” and “drugs” are difficult to be separated in the short run, the inhibitory effects of buyer power on the economic effect and behavioral decisions of pharmaceutical companies will remain in place for a longer period. The influence of horizontal market factors on pharmaceutical companies changes with the external environment. In the face of strong buyers, pharmaceutical companies could boost their counterbalance power through a variety of channels. The short-run policies are as follows: (1) With respect to supply chain management, pharmaceutical companies should optimize their relationships with downstream customers in the industrial chain, both to preserve the stability of partners and to expand the range of external options for downstream customers to develop a diversified relationship network of suppliers-customers. (2) The pharmaceutical enterprises not only need to rely on continuous investment in research and development to maintain its technological advantage but also must adopt effective market strategies and product innovations to maintain its market shares. At the same time, while pursuing short-term returns, pharmaceutical enterprises should place greater emphasis on the sustainability of future profit growth and their long-term production and management capacity, in order to cope with fierce horizontal market competition as well as vertical market competition from downstream customers. (3) With respect to vertical cooperation, pharmaceutical companies should establish new information shared mechanisms of teamwork, risk-sharing, and mutually-supported with large customers, extend and strengthen the industry chain, enhance the level of trust between both parties, so as to mitigate the negative effects of buyers on suppliers' profits. (4) With respect to negotiation skills and public relations, pharmaceutical companies should establish professional negotiation teams and devise differentiated negotiation strategies for different downstream customers, so as to strategically cushion the adverse effects of their inherent weaknesses relative to the healthcare industry in market transactions, with a view to securing more benefits in market transactions.

The long-run policies are as follows: (1) With respect to corporate management, pharmaceutical companies should strengthen the development of their independent innovation capabilities and gain competitive advantages through product and technological innovation in a fiercely competitive market environment, with the aim of enhancing their performance. Pharmaceutical companies should improve their governance structure, optimize resource allocation, and implement diverse strategies for innovation investment, in order to enhance their competitive advantages in the market. (2) With respect to product production, pharmaceutical companies should actively adjust their development strategies to adapt to changes in market demand, focus on a specific area of better medicine production (safer, more health benefits, more cost-effective with lower manufacturing costs), and enhance their bargaining power in market transactions through value-adding products and services. (3) With respect to market support and regulation, the government should build a comprehensive innovation system, such as by increasing innovation investment in basic research of pharmaceutical companies and assisting pharmaceutical companies in their innovative R&D. The government should formulate targeted and differentiated subsidy policies based on the R&D situations of different types of pharmaceutical companies and their market positions in the industrial chain, with the aim of guiding companies to optimize the efficiency of their R&D investment.

## 6 Discussion

The findings of this study not only provide theoretical foundations and practical approaches for regulating large buyer groups (cartels) in vertical market transactions, but also assist regulatory authorities in identifying and preventing potential market risks, thereby safeguarding fairness and efficiency in market competition. At the same time, the results help promote more balanced interactions among upstream and downstream enterprises in the pharmaceutical industry chain, and enhance the overall stability and resilience of the industry chain. Against the backdrop of multiple uncertainties facing the global pharmaceutical supply chain, this research offers important insights for policymakers and business leaders to optimize governance mechanisms and strengthen risk resistance capabilities.

## Data Availability

The original contributions presented in the study are included in the article/Supplementary material, further inquiries can be directed to the corresponding author.
