# Supplementary material for: Study on the relationship between buyer market power, spatial spillover effect, and profit in China's pharmaceutical industry
Source: Front Public Health. 2025 Nov 14;13:1683074. doi: 10.3389/fpubh.2025.1683074 (PMC12660219; doi:10.3389/fpubh.2025.1683074)
Supplement: Supplementary file 1 [file Presentation_1.pdf]

Appendix

Direct effect, indirect effect, and total effect

After the first item on the right side of the equal sign of Equation (1) is moved to the left side of the equal sign, the following equation can be obtained:

$$(\boldsymbol{I_n} - \rho \boldsymbol{W})\boldsymbol{Y} = \boldsymbol{\beta X} + \boldsymbol{\delta W X} + \boldsymbol{\varepsilon} \tag{2}$$

In the equation, $\boldsymbol{I_n}$ stands for the $n \times n$ dimensional unit matrix, $\boldsymbol{Y}$ for the vector of the dependent variable, and $\boldsymbol{X}$ for a matrix consisting of independent variable and control variable. To determine the direct effect, indirect effect, and total effect, Equation (2) is multiplied by $(\boldsymbol{I_n} - \rho \boldsymbol{W})^{-1}$ on both sides to generate the following equation:

$$\boldsymbol{Y} = \sum\nolimits_{r=1}^{k} \boldsymbol{S_r(W)X_r} + \boldsymbol{V(W)\varepsilon} \tag{3}$$

In the equation, $\boldsymbol{S_r(W)} = \boldsymbol{V(W)}(\beta_r \boldsymbol{I_n} + \delta_r \boldsymbol{W})$ 、 $\boldsymbol{V(W)} = (\boldsymbol{I_n} - \rho \boldsymbol{W})^{-1} = \boldsymbol{I_n} + \rho \boldsymbol{W} + \rho^2 \boldsymbol{W}^2 + \rho^3 \boldsymbol{W}^3 + \cdots$. Suppose that $\boldsymbol{X}$ includes k explanatory variables, then the r −th explanatory variable is $\boldsymbol{X_r} = (\boldsymbol{x_{1r}}, \boldsymbol{x_{2r}}, \cdots, \boldsymbol{x_{nr}})^T$; β_r stands for the coefficient of $\boldsymbol{I_n}$ and δ_r for the coefficient of $\boldsymbol{W}$. After Equation (3) is extended, the following equation can be obtained:

$$\begin{pmatrix} \boldsymbol{Y_1} \\ \boldsymbol{Y_2} \\ \vdots \\ \boldsymbol{Y_n} \end{pmatrix} = \sum\nolimits_{r=1}^{k} \begin{pmatrix} \boldsymbol{S_r(W)_{11}} & \boldsymbol{S_r(W)_{12}} & \cdots & \boldsymbol{S_r(W)_{1n}} \\ \boldsymbol{S_r(W)_{21}} & \boldsymbol{S_r(W)_{22}} & \cdots & \boldsymbol{S_r(W)_{2n}} \\ \vdots & \vdots & \vdots & \vdots \\ \boldsymbol{S_r(W)_{n1}} & \boldsymbol{S_r(W)_{n2}} & \cdots & \boldsymbol{S_r(W)_{nn}} \end{pmatrix} \begin{pmatrix} \boldsymbol{x_{1r}} \\ \boldsymbol{x_{2r}} \\ \vdots \\ \boldsymbol{x_{nr}} \end{pmatrix} + \boldsymbol{V(W)\varepsilon} \tag{4}$$

The partial differential matrix of the explained variable $\boldsymbol{Y}$ on $\boldsymbol{X_r}$ at a specific moment is as follows:

$$\left(\frac{\partial \boldsymbol{Y}}{\partial \boldsymbol{x_{1r}}} \frac{\partial \boldsymbol{Y}}{\partial \boldsymbol{x_{2r}}} \cdots \frac{\partial \boldsymbol{Y}}{\partial \boldsymbol{x_{nr}}}\right) = \begin{pmatrix} \frac{\partial \boldsymbol{y_1}}{\partial \boldsymbol{x_{1r}}} & \frac{\partial \boldsymbol{y_1}}{\partial \boldsymbol{x_{2r}}} & \cdots & \frac{\partial \boldsymbol{y_1}}{\partial \boldsymbol{x_{nr}}} \\ \vdots & \ddots & & \vdots \\ \frac{\partial \boldsymbol{y_n}}{\partial \boldsymbol{x_{1r}}} & \cdots & & \frac{\partial \boldsymbol{y_n}}{\partial \boldsymbol{x_{1r}}} \end{pmatrix} = (\boldsymbol{I_n} - \rho \boldsymbol{W})^{-1} \begin{pmatrix} \beta_r & W_{12}\delta_r & \cdots & W_{1n}\delta_r \\ W_{21}\delta_r & \beta_r & \cdots & W_{2n}\delta_r \\ \vdots & \vdots & \ddots & \vdots \\ W_{2n}\delta_r & \cdots & \cdots & \beta_r \end{pmatrix} \tag{5}$$

From equation (5), we can infer that the r-th independent variable in region i influences both the local dependent variable and the dependent variables across all regions, including those in other regions. $\bar{M}(r)_{direct}$ is set as the average direct effect, which indicates the average effect of the r-th explanatory variable in the i region on the local dependent variable $\boldsymbol{Y_i}$, $\bar{M}(r)_{direct} = n^{-1} trace[\boldsymbol{S_r(W)}]$; $\bar{M}(r)_{total}$ is the average total effect, representing the average effect of the r-th explanatory variable in the i region on all regional dependent variables $\boldsymbol{Y_j}$, $\bar{M}(r)_{total} = n^{-1} \boldsymbol{i_n^T S_r(W) i_n}$, $\boldsymbol{i_n} = (1, 1, \cdots, 1)^T_{1\times n}$ and T is the vector matrix transposition; $\bar{M}(r)_{indirect}$ is the average indirect effect, and $\bar{M}(r)_{indirect} = \bar{M}(r)_{total} - \bar{M}(r)_{direct}$ represents the difference between the average total effect and the average direct effect.

Attached Table 1 The Unit, Symbol and Definition of Each Variable

Variable	*unit*	*Symbol*	*definition*
Profit	100 million	Profit	the annual net profit of the regional pharmaceutical industry
Buyer Power	number	Bmp	the total number of hospitals in a province
Seller power1	number	Smp_1	the total number of pharmaceutical enterprises in a province
Seller power2	100 million	Smp_2	the total assets of pharmaceutical industry in a province
Seller power3	100 million	Smp_3	the total industrial output value of pharmaceutical industry in a province
Buyer power × Seller power	number × number	Bmp • Smp	the total number of hospitals in a province × the total number of pharmaceutical enterprises in a province
Enterprise size	100 million	Size	dividing the total industrial output value of the pharmaceutical sector by the number of pharmaceutical companies
Asset specificity	%	Asset	the ratio of fixed asset investment in the pharmaceutical industry relative to the total asset investment at the end of the year
R&D investment	%	Rnd	the ratio of internal R&D spending to sales revenue
Barriers to entry	100 million	Barrier	The mount of fixed asset investment of pharmaceutical industry in a province
Government price regulation	%	Govr	ex - factory price index of pharmaceutical industry divided by ex - factory price index of the general industrial products in a province
Market demand growth rate	%	Demand	the difference between the current sales revenue and the previous sales revenue divided by the current sales revenue.
Per capita GDP	100 hundred	Reg	Per capita GDP
Return on assets	%	Roa	net profits divided by total assets
Capital density	100 million	Capital	dividing fixed asset investments by the number of employees
Density of R&D personnel	%	Ratio	the ratio of the number of R&D personnel to the total number of employees
Number of new products developed	number	Items	the number of new product development projects

Attached Table 2 Descriptive Statistics of Each Variable

Variable	*Obs*	*Mean*	*St*	*Min*	*Max*
Profit	651	38.762	62.110	0.130	508.970

Bmp	651	684.569	388.318	93.000	2378.000
Smp_1	651	245.631	170.728	3.000	969.0000
Smp_2	651	368.655	454.983	5.770	3523.260
Smp_3	651	420.975	516.204	3.010	4851.323
Bmp • Smp_1	651	173582.000	230649.700	428.000	2.176e+06
Bmp • Smp_2	651	403715.200	1.005e+06	232.260	2.450e+07
Bmp • Smp_3	651	402177.500	819352.000	281.790	9.208e+06
Size	651	1.396	1.048	0.009	5.169
Asset	651	0.370	0.074	0.122	0.760
Rnd	651	0.016	0.007	2.610e-05	0.056
Barrier	651	112.343	144.456	1.320	1334.972
Govr	651	0.960	0.043	0.832	1.182
Demand	651	0.217	0.128	-0.289	1.137
Reg	651	3.154	2.453	0.290	13.729
Roa	651	0.056	0.037	0.001	0.274
Capital	651	0.002	0.001	0.0006	0.012
Ratio	651	0.056	0.037	0.001	0.274
Items	651	417.393	565.660	1.000	4716.000

Attached Table 3 Cluster Standard Error Regressions for the OLS

Explanatory Variable	*Profit*		
	Coefficient		
Buyer Power	-0.016*** (-3.500)	-0.040*** (-4.950)	-0.031*** (-3.08)
Seller power1	0.088*** (5.970)		
Buyer Power • Seller power1	1.457*** (10.050)		
Seller power2		0.073*** (3.320)	
Buyer Power • Seller power2		0.623*** (9.660)	
Seller power3			0.034** (2.240)
Buyer Power • Seller power3			0.132** (2.050)
Enterprise size	4.122** (2.186)	5.399*** (3.398)	4.691** (2.364)

Asset specificity	-168.816* (-1.788)	-149.183** (-2.196)	-140.535* (-1.831)
Barriers to entry	0.210*** (9.450)	0.035*** (3.930)	0.261*** (5.590)
Government price regulation	-5.542 (-0.43)	-4.713 (-0.380)	-6.026 (-0.420)
Market demand	1.680 (0.450)	4.244 (1.150)	0.207 (0.050)
Per capita GDP	4.550*** (8.180)	4.925*** (7.380)	2.908*** (4.500)
Return on assets	228.535* (1.765)	209.311* (1.913)	171.183 (1.290)
Capital density	7201.458** (2.339)	6179.204* (1.795)	5765.838 (1.225)
Density of R&D personnel	75.340*** (4.160)	46.466*** (3.630)	69.810*** (2.920)
New products	0.018*** (4.170)	0.002** (2.170)	0.021*** (4.150)
R^2	0.949	0.962	0.938
N	496	496	496

Attached Table 4 Robustness Test of Seller Power Based on the Total Assets of the Pharmaceutical Industry

Panel A ***W1***				
Explanatory Variable	*Profit*			
	Direct effect	*Indirect effect*	*Total effect*	*Pool regression*
Buyer power	-0.057***	-0.010	-0.067**	-0.050***
	(-4.896)	(-0.870)	(-2.392)	(-4.800)
Seller power2	0.051***	0.066	0.117**	0.051***
	(7.462)	(1.333)	(2.283)	(7.329)
Buyer Power • Seller power2	0.404***	-0.653***	-0.250	0.401***
	(12.292)	(-2.826)	(-1.046)	(12.355)
Control Variables	Control	Control	Control	Control
W1 *• Bmp*	-0.0566 (-0.574)			
R^2	0.966			
Maximum likelihood	-1916.285			
Panel B ***W2***				

Explanatory Variable	*Profit*			
	Direct effect	Indirect effect	Total effect	Pool regression
Buyer power	-0.088***	-0.020	-0.108***	-0.086***
	（-4.436）	（-0.100）	（-3.538）	（-4.319）
Seller power2	0.051***	0.131***	0.182***	0.052***
	（7.246）	（2.753）	（3.627）	（7.137）
Buyer Power • *Seller power2*	0.380***	-0.808***	-0.427*	0.376***
	（11.395）	（-3.579）	（-1.811）	（11.400）
Control Variables	Control	Control	Control	Control
W2 • *Bmp*	-0.084			
	（-0.317）			
R^2	0.967			
Maximum likelihood	-1915.592			

Attached Table 5 Robustness Test of Seller Power Based on the total industrial output value of the pharmaceutical industry

Panel A ***W1***

Explanatory Variable	*Profit*			
	Direct effect	*Indirect effect*	*Total effect*	*Pool regression*
Buyer power	-0.054**	-0.005	-0.058**	-0.049**
	（-2.603）	（-0.198）	（-2.673）	（-2.208）
Seller power3	0.033***	0.004	0.037	0.031***
	（2.767）	（1.112）	（1.247）	（2.829）
Buyer Power • *Seller power3*	0.106***	0.643***	0.750**	0.102***
	（3.451）	（2.747）	（2.242）	（3.351）
Control Variables	Control	Control	Control	Control
W1 • *Bmp*	-0.059			
	（-0.569）			
R^2	0.955			
Maximum likelihood	-1983.935			

Panel B ***W2***

Explanatory Variable	*Profit*			
	Direct effect	Indirect effect	Total effect	Pool regression
Buyer power	-0.079***	-0.001	-0.080***	-0.080***
	（-4.024）	（-0.440）	（-3.978）	（-3.501）
Seller power3	0.003**	0.010**	0.013**	0.003***
	（2.252）	（2.336）	（2.237）	（3.378）

Buyer Power • *Seller power3*	0.141*** （4.603）	0.177 （0.909）	0.317 （0.181）	0.138*** （4.511）
Control Variables	Control	Control	Control	Control
**W2** *• Bmp*		-0.076 （-1.125）		
R^2		0.955		
Maximum likelihood		-1986.3398		
